# Analysis of Hereditary Elliptocytosis with Decreased Binding of Eosin-5-maleimide to Red Blood Cells

**DOI:** 10.1155/2015/451861

**Published:** 2015-10-18

**Authors:** Shin-ichiro Suemori, Hideho Wada, Hidekazu Nakanishi, Takayuki Tsujioka, Takashi Sugihara, Kaoru Tohyama

**Affiliations:** ^1^Department of Laboratory Medicine, Kawasaki Medical School, Kurashiki, Japan; ^2^Department of Hematology, Kawasaki Medical School, Kurashiki, Japan

## Abstract

Flow cytometric test for analyzing the eosin-5-maleimide (EMA) binding to red blood cells has been believed to be a specific method for diagnosing hereditary spherocytosis (HS). However, it has been reported that diseases other than HS, such as hereditary pyropoikilocytosis (HPP) and Southeast Asian ovalocytosis (SAO), which are forms in the category of hereditary elliptocytosis (HE), show decreased EMA binding to red blood cells. We analyzed EMA binding to red blood cells in 101 healthy control subjects and 42 HS patients and obtained a mean channel fluorescence (MCF) cut-off value of 36.4 (sensitivity 0.97, specificity 0.95). Using this method, we also analyzed 12 HE patients. Among them, four HE patients showed the MCF at or below the cut-off value. It indicates that some HE patients have decreased EMA binding to red blood cells. Two of these four HE patients were classified as common HE, and two were spherocytic HE with reduced spectrin. This study demonstrates that, in addition to patients with HPP or SAO, some HE patients have decreased EMA binding to red blood cells.

## 1. Introduction

Eosin-5-maleimide (EMA) is a fluorochrome that primarily binds to band 3 of red blood cell membrane proteins. EMA binding decreases in hereditary spherocytosis (HS), which is considered to be a useful finding for the diagnosis of HS [1–6]. Moreover, among patients with red blood cell membrane abnormalities other than HS, some patients with hereditary pyropoikilocytosis (HPP) and Southeast Asian ovalocytosis (SAO), which are forms of hereditary elliptocytosis (HE), also show decreased EMA binding to red blood cells [7, 8]. The etiology of HE includes abnormalities in membrane proteins involved in formation of the membrane structure, including spectrin, protein 4.1 (P4.1), and glycophorin C. HE is basically classified into 5 forms: common HE, spherocytic HE, HPP, SAO, and HE with X chromosome abnormality, based on differences in pathological conditions [9]. There have been few studies examining EMA binding to red blood cells in the HE patients, other than HPP and SAO, and the results are variable [1, 3–5]. We analyzed EMA binding to red blood cells in 12 HE patients observed in our department and examined the relationship between the types of HE and EMA binding to red blood cells.

## 2. Subjects and Methods

All red blood cells were obtained following informed consent. And all patients gave written informed consent. The study involved 12 HE and 42 HS patients examined in our department between December 2008 and June 2012; 101 healthy subjects were used as a control group. A diagnosis of HS and HE was based on a complete blood count, biochemical analysis, family analysis, red blood cell morphology using scanning electron microscopy (SEM), red blood cell membrane protein analysis using sodium dodecyl sulfate-polyacrylamide gel electrophoresis (SDS-PAGE), and EMA binding to red blood cells. The analyses of all 12 HE patients were requested by other institutions. And the osmotic fragility test was not performed in any patients at client institutions. This study was approved by the Research Ethics Committee of Kawasaki Medical School and Hospital.

### 2.1. Evaluation of Peripheral Red Blood Cell Morphology

Fresh venous blood was drawn to evaluate red blood cell morphology from peripheral blood. A sample was fixed with 0.1 M phosphate buffer with 1% glutaraldehyde (pH 7.4) and observed using a scanning electron microscope (S-3400N, HITACHI High-Technologies Corporation). HE can be largely classified into either rod-shaped or ovalocytic based on differences in the degree of red blood cell ovalization. In this study, red blood cells with long diameter/short diameter ≥2 were defined as rod-shaped, those with long diameter/short diameter <2 were defined as ovalocytic, and the percentages of the rod-shaped and ovalocytic types in 100 randomly observed red blood cells were calculated.

### 2.2. Analysis of EMA Binding to Red Blood Cells

Based on the original method of King et al. [1], red blood cells were washed with phosphate buffer saline (PBS) four times in a microtube; after that, 5 volumes of EMA (5 mg/mL) were added to 1 volume of packed red blood cells, and the sample was mixed well. It was then incubated for 1 hour at room temperature in the dark to allow EMA to bind to the red blood cells. After EMA binding, the sample was centrifuged at 13,000 rpm for 10 seconds, the supernatant was removed, and the sample was washed with 0.5% bovine serum albumin (BSA)/PBS. After repeating this procedure three times, the sample was diluted in a 0.5% BSA/PBS in a final ratio of 14 : 1 packed red blood cells. Thereafter, flow cytometry was performed using the FL-1 channel at an event count of 15,000 using a FACSCalibur Flow Cytometer (Becton Dickinson), and fluorescence intensity values were obtained as mean channel fluorescence (MCF). MCF was measured three times for each sample, and the mean value was used.

### 2.3. Preparation of Red Blood Cell Ghost

Red blood cell ghosts were prepared according to the method of Dodge et al. [10]. Ethylenediaminetetraacetic acid (EDTA) and phenylmethylsulfonyl fluoride (PSMF) were added as protease inhibitors to lysis buffer (5 mmol/L Na_2_HPO_4_, 1 mmol/L EDTA, and 0.2 mmol/L PMSF [pH 8.0]). After washing three times with 0.9% NaCl, 30 volumes of lysis buffer were added to 1 volume of packed cells, which was allowed to stand for 5 minutes at 4°C to perform hypotonic hemolysis. Then, the sample was centrifuged at 30,000 ×g for 10 minutes at 4°C to separate red blood cell membrane protein and hemoglobin; and the hemoglobin component in the supernatant was removed by aspiration. These steps were repeated six to seven times to extract the red blood cell white ghost, which was used as a red blood cell membrane protein sample for subsequent examinations.

### 2.4. Separation and Evaluation of Red Blood Cell Membrane Protein

Each red blood cell membrane protein fraction was separated by SDS-PAGE using the Fairbanks technique [11] (3.5% to 17% exponential gradient gel: acrylamide = 35 : 8). After SDS-PAGE, each membrane protein fraction was identified by staining the gel with Coomassie brilliant blue R-250. After the gel was dried, the absorbance of each band was measured using densitometer (GS-800 Calibrated Densitometer, Bio-Rad). Then, results were analyzed by Quantity One software (Bio-Rad). Each band, representing a membrane protein fraction, is presented as a ratio to the combined density of the total membrane fraction (bands 1–7). Membrane protein deficiency was determined when patients had ≥10% and ≥2 standard deviation difference relative to the mean stain density for each fraction in healthy control subjects (*n* = 3); controls were run on the same gel as the study subjects. Protein concentration was measured at 655 nm using the DC Protein Assay Kit (Bio-Rad) based on the Lowry technique, and 25 *μ*g of red blood cell membrane protein was used for electrophoresis [12].

### 2.5. Statistical Analysis

Significant differences were tested by the Mann-Whitney *U* test. Analysis and construction of receiver operating characteristic (ROC) curves were accomplished using SPSS Statistics version 20.0 (IBM).

## 3. Results

### 3.1. Analysis of EMA Binding to Red Blood Cells in Healthy Subjects, HS Patients, and HE Patients

**Figure 1:**
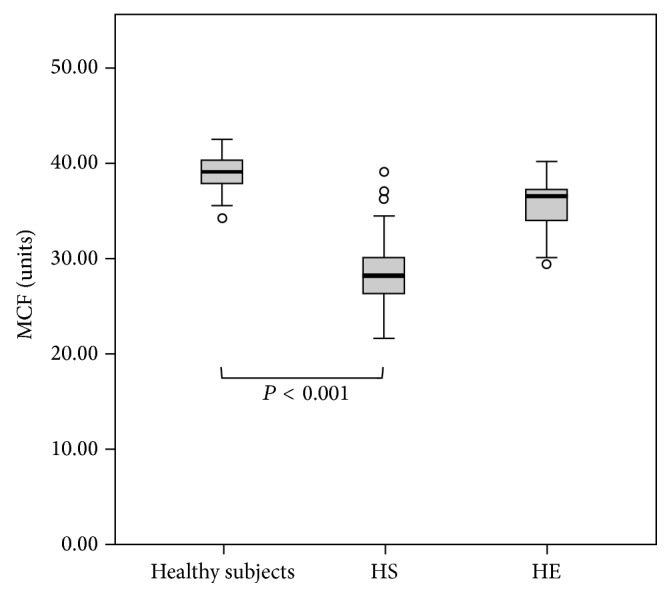
EMA binding in healthy subjects, HS patients, and HE patients. MCF in healthy subjects = 39.2 ± 1.6 (*n* = 101), MCF in HS patients = 28.8 ± 3.8 (*n* = 42), and MCF in HE patients = 35.6 ± 3.3 (*n* = 12) (MCF cut-off value: 36.4).

Using ROC curves of MCF values for EMA binding to red blood cells, the cut-off value in HS patients was 36.4 (sensitivity 0.97, specificity 0.95). MCF values below this value were defined as decreased EMA binding to red blood cells. Four of 12 HE patients showed decreased EMA binding (Figure 1, Table 1).

### 3.2. Association between EMA Binding and HE Types (Table 1)

**Figure 2:**
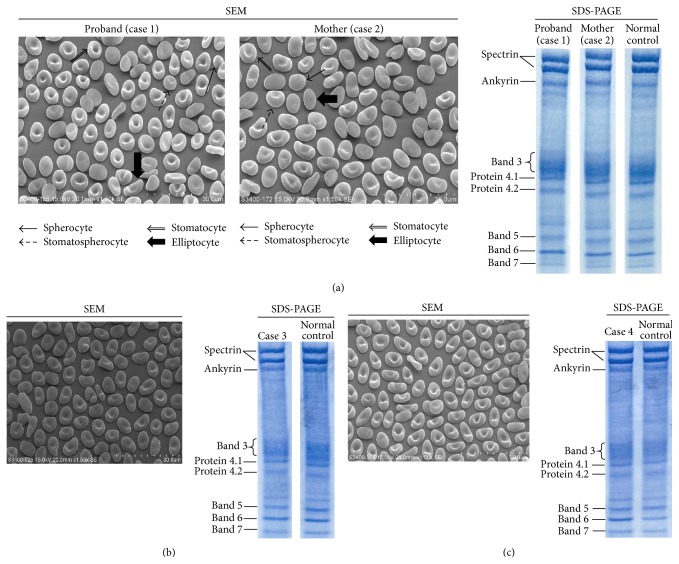
SEM images and results of SDS-PAGE gels of HE patients with decreased EMA binding to red blood cells. (a) SEM image and SDS-PAGE of Family A (case 1, case 2). SDS-PAGE: proband (case 1): spectrin 14.2% reduction, mother (case 2): spectrin 14.5% reduction. (b) SEM image and SDS-PAGE of case 3. SEM: ovalocytic type and rod-shaped type were observed. SDS-PAGE: no membrane protein abnormality detected. (c) SEM image and SDS-PAGE of case 4. SEM: many rod-shaped types were observed. SDS-PAGE: no membrane protein abnormality detected.

Based on SEM findings, elliptocytes were present in addition to the findings observed in HS such as spherocyte development and decreased spectrin in cases 1 and 2. So these cases were diagnosed with spherocytic HE, not HS (Figure 2(a)). These two cases demonstrated decreased EMA binding to red blood cells.

SEM findings also showed that 10 other HE patients had common HE. Among them, two showed decreased EMA binding (Figures 2(b) and 2(c)).

### 3.3. Association between EMA Binding and Red Blood Cell Membrane Protein Abnormalities in HE Patients (Table 1)

Red blood cell membrane protein analysis in 12 HE patients indicated that two had partial spectrin deficiency and five had partial P4.1 deficiency. Two patients with partial spectrin deficiency (cases 1 and 2) showed decreased EMA binding (Figure 2(a)). No patients with partial P4.1 deficiency showed decreased EMA binding. In addition, two patients (cases 3 and 4) clearly showed elliptocytosis with decreased EMA binding to red blood cells. However, no abnormalities in membrane proteins (Figures 2(b) and 2(c)) were detected.

No significant decrease in band 3 was detected by SDS-PAGE in four patients with decreased EMA binding.

### 3.4. Association between EMA Binding and Red Blood Cell Ovalization in HE Patients

**Figure 3:**
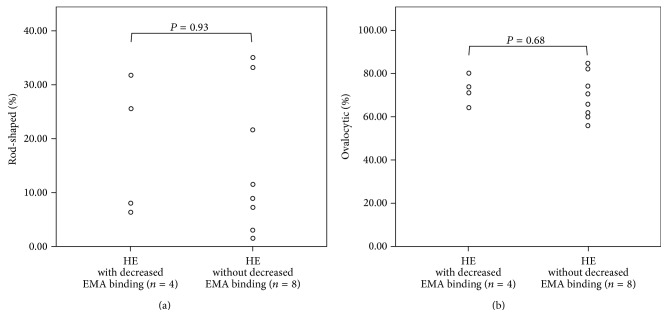
Association between the presence/absence of decreased EMA binding and elliptocyte development in HE patients. (a) Association between the presence/absence of decreased EMA binding and the percentage of rod-shaped type in HE patients. The percentage of rod-shaped type in 4 HE patients with decreased EMA binding = 18.0 ± 12.7%. The percentage of rod-shaped type in 8 HE patients without decreased EMA binding = 15.3 ± 13.2%. Rod-shaped type: RBC long diameter/short diameter ≥2. (b) Association between the presence/absence of decreased EMA binding and the percentage of ovalocytic type in HE patients. The percentage of the ovalocytic type in 4 HE patients with decreased EMA binding = 72.2 ± 6.6%. The percentage of the ovalocytic type in 8 HE patients without decreased EMA binding = 69.4 ± 10.4%. Ovalocytic type: RBC long diameter/short diameter <2.

Red blood cells with long diameter/short diameter ≥2 were defined as rod-shaped, and those with long diameter/short diameter <2 were defined as ovalocytic. The percentages of the rod-shaped and ovalocytic types in 100 red blood cells on SEM images were calculated. The results showed that the rod-shaped type comprised 18.0% ± 12.7% in four HE patients with decreased EMA binding and 15.3% ± 13.2% in eight HE patients with normal EMA binding. Therefore, no clear correlation was observed between decreased EMA binding and the ratio of rod-shape type (Figure 3(a)). The ovalocytic type comprised 72.2% ± 6.6% in four HE patients with decreased EMA binding and 69.4% ± 10.4% in eight HE patients with normal EMA binding. Thus, these results indicate no clear correlation between decreased EMA binding and the ratio of the ovalocytic type (Figure 3(b)).

## 4. Discussion

In the present study, four HE patients demonstrated decreased EMA binding. Among them, two were spherocytic HE with spectrin abnormalities. Spectrin abnormalities can also cause HS as well as HE. For example, genetic mutations mainly causing decreased spectrin are common in HS [13], whereas mutations causing abnormalities in spectrin self-association are common in HE [14]. These two patients had partial spectrin deficiency, as observed in some patients with HS. Spherocytic HE has the features of both HS and HE. So it is suggested that the decreased EMA binding observed in two spherocytic HE patients might have exhibited the features of HS.

In this study, decreased EMA binding to red blood cells was observed in two patients with common HE in whom no membrane protein abnormalities were detected by SDS-PAGE.

Considering various degrees of red blood cell ovalization among HE patients, we examined differences in the trend of ovalization as factors involved in decreased EMA binding. However, no association was observed between the degree of ovalization and EMA binding. The mechanism underlying the decrease in EMA binding to red blood cells in these patients is unknown.

This study revealed that some HE patients, other than those with HPP or SAO, present with decreased EMA binding to red blood cells. The EMA binding test is believed to be a specific method for diagnosing HS. However, it should be noted that decreased EMA binding can be observed even in some HE patients similarly to HS patients. Historically, congenital hemolytic anemia induced by red cell membrane abnormalities was defined based on their characteristic red blood cell shape [15]. The HS guidelines revised in 2011 [16] show that the EMA binding test is useful for screening atypical HS patients. However, attention should be paid to the presence of patients with spherocytic HE or common HE with decreased EMA binding.

## Figures and Tables

**Table 1:** List of HE Patients.

Case	Family	HE type	Membrane protein abnormality (SDS-PAGE)	Spectrin patient/control (%) (SDS-PAGE)	Protein 4.1 patient/control (%) (SDS-PAGE)	Band 3 patient/control (%) (SDS-PAGE)	Rod-shaped RBC (%)	Ovalocytic RBC (%)	MCF (units)	Hb (g/dL)	Haptoglobin (mg/dL)	ID-Bil (mg/dL)	Reticulo (/*μ*L)	MCV (fL)	MCHC (%)
1	A-1	Spherocytic HE	Partial spectrin deficiency	85.8	103.2	106.7	31.8	64.2	32.4	4.7	2	1.3	12.3	74.3	36.2
2	A-2	Spherocytic HE	Partial spectrin deficiency	85.5	95.1	107.5	8.0	80.0	30.2	Not tested	2	1.4	Not tested	Not tested	Not tested
3	B	Common HE	Membrane protein abnormality not detected	93.3	99.4	102.4	6.4	73.8	29.2	12.2	<8	1.3	25.7	86.3	35.2
4	C	Common HE	Membrane protein abnormality not detected	103.7	92.3	99.7	25.6	70.9	35.7	11.8	<2	0.9	8.6	89.6	33.5
5	D-1	Common HE	Membrane protein abnormality not detected	90.4	101.2	103.3	7.2	84.6	37.3	12.3	4	0.9	10.5	81.5	34.5
6	D-2	Common HE	Membrane protein abnormality not detected	92.4	104.0	104.9	11.5	82.2	36.6	12.2	18	1.3	7.7	75.8	35.5
7	D-3	Common HE	Membrane protein abnormality not detected	90.3	102.8	102.8	3.0	65.8	38.7	13.6	44	0.3	4.5	88.2	34.3
8	E-1	Common HE	Partial P4.1 deficiency	94.8	76.5	100.0	21.6	70.6	37.2	12.3	<2	0.5	2.7	82.7	33.3
9	E-2	Common HE	Partial P4.1 deficiency	102.9	79.0	99.5	1.5	60.0	36.2	15.6	67	1.2	6.1	86.8	35.5
10	E-3	Common HE	Partial P4.1 deficiency	102.9	76.2	97.9	9.0	74.1	37.2	14.7	32	0.8	6.7	91.9	35.9
11	F	Common HE	Partial P4.1 deficiency	100.9	75.4	99.0	35.1	55.9	36.7	13.2	<2	0.5	8.3	86	35.1
12	G	Common HE	Partial P4.1 deficiency	99.8	82.2	110.6	33.3	61.7	40.3	10.7	<5	0.5	6	81	30.7

MCF cut-off value: 36.4; reference value: haptoglobin (19–170 mg/dL).
